# Reliability of a novel thermal imaging system for temperature assessment of healthy feet

**DOI:** 10.1186/s13047-018-0266-1

**Published:** 2018-05-30

**Authors:** N. L. Petrova, A. Whittam, A. MacDonald, S. Ainarkar, A. N. Donaldson, J. Bevans, J. Allen, P. Plassmann, B. Kluwe, F. Ring, L. Rogers, R. Simpson, G. Machin, M. E. Edmonds

**Affiliations:** 1 Diabetic Foot Clinic, King’s College Hospital NHS Foundation Trust, Denmark Hill, London, SE5 9RS UK; 2 Division of Diabetes and Nutritional Sciences, King’s College London, London, UK; 3 Temperature and Humidity, National Physical Laboratory, London, UK; 4 Microvascular Diagnostics, Northern Medical Physics and Clinical Engineering, Newcastle upon Tyne Hospitals, Newcastle upon Tyne, UK; 5 Community Podiatry Department, Pennine Acute Hospitals NHS Trust, Manchester, UK; 6 Photometrix Imaging Ltd, Pontypridd, UK; 7 Department of Computing, University of South Wales, Pontypridd, UK

**Keywords:** Thermal imaging, Diabetic foot ulcer, Temperature, Reliability, Prevention

## Abstract

**Background:**

Thermal imaging is a useful modality for identifying preulcerative lesions (“hot spots”) in diabetic foot patients. Despite its recognised potential, at present, there is no readily available instrument for routine podiatric assessment of patients at risk. To address this need, a novel thermal imaging system was recently developed. This paper reports the reliability of this device for temperature assessment of healthy feet.

**Methods:**

Plantar skin foot temperatures were measured with the novel thermal imaging device (Diabetic Foot Ulcer Prevention System (DFUPS), constructed by Photometrix Imaging Ltd) and also with a hand-held infrared spot thermometer (Thermofocus® 01500A3, Tecnimed, Italy) after 20 min of barefoot resting with legs supported and extended in 105 subjects (52 males and 53 females; age range 18 to 69 years) as part of a multicentre clinical trial. The temperature differences between the right and left foot at five regions of interest (ROIs), including 1st and 4th toes, 1st, 3rd and 5th metatarsal heads were calculated. The intra-instrument agreement (three repeated measures) and the inter-instrument agreement (hand-held thermometer and thermal imaging device) were quantified using intra-class correlation coefficients (ICCs) and the 95% confidence intervals (CI).

**Results:**

Both devices showed almost perfect agreement in replication by instrument. The intra-instrument ICCs for the thermal imaging device at all five ROIs ranged from 0.95 to 0.97 and the intra-instrument ICCs for the hand-held-thermometer ranged from 0.94 to 0.97. There was substantial to perfect inter-instrument agreement between the hand-held thermometer and the thermal imaging device and the ICCs at all five ROIs ranged between 0.94 and 0.97.

**Conclusions:**

This study reports the performance of a novel thermal imaging device in the assessment of foot temperatures in healthy volunteers in comparison with a hand-held infrared thermometer. The newly developed thermal imaging device showed very good agreement in repeated temperature assessments at defined ROIs as well as substantial to perfect agreement in temperature assessment with the hand-held infrared thermometer. In addition to the reported non-inferior performance in temperature assessment, the thermal imaging device holds the potential to provide an instantaneous thermal image of all sites of the feet (plantar, dorsal, lateral and medial views).

**Trial registration:**

Diabetic Foot Ulcer Prevention System NCT02317835, registered December 10, 2014

## Background

Diabetic foot ulcer is a major complication of diabetes [1]. In people with diabetic neuropathy, tissue damage can progress to ulcer, infection and necrosis and ultimately results in amputation [2]. Indeed, in diabetes, almost 85% of all non-traumatic amputations are preceded by a foot ulcer. The financial cost of foot ulcers and amputations is immense [3, 4]. Diabetic foot ulcer imposes substantial burden on public and private payers, ranging from $9–13 billion in addition to the costs associated with diabetes itself [3]. A recent health economics analysis has reported that the total expenditure on healthcare related to foot ulcer and amputation in people with diabetes for 2014–2015 in England was estimated at £1billion [4]. At least £1 in every £140 of the National Health Service (NHS) expenditure in England is spent on footcare for people with diabetes [4]. This is equivalent to around 0.7–0.8% of the entire NHS budget. Recent data show that at least 60,671–75,838 people with diabetes in England have foot ulcers at any given time (2–2.5% of the diagnosed diabetes population), and that the mean weekly cost of caring for each patient is £208 [4]. Thus timely identification of patients at risk is fundamental to reduce adverse outcomes and reduce costs [5, 6]. It has been estimated that reducing the prevalence of people with diabetic foot ulcers by one third could save the NHS £210 m–£262 m a year [4].

Over the last fifteen years there has been an increased interest in thermal imaging as a possible modality for early detection of incipient tissue damage in diabetic foot patients [7–9]. Clinical trials have indicated that regular measurement of foot skin temperatures with non-contact infrared thermometers in high-risk patients can reduce the incidence of foot ulcers [10]. However, in these studies, foot temperatures were assessed only at predefined regions of interest (ROIs) using single spot infrared thermometers [10, 11] and the low specificity of this method is now well recognised [12]. Thus, despite the evidence that neuropathic foot ulcer is preceded by a rise in skin temperature [11] the latter is not routinely measured in clinical practice.

There is a requirement for a reliable portable device as certified to medical device regulations to document thermal images of high risk diabetic foot patients during routine podiatry assessment. The ideal thermal imager should be user friendly, widely available, reproducible and accurate [13]. In addition, thermal imaging should not only be limited to the plantar site of the feet as more than half of the diabetic foot ulcers (52%) are with non-plantar location [14]. Detailed assessment with such a device can provide information of up to several thousand ROIs as opposed to up to 12 ROIs most commonly assessed by podiatrists using non-contact infrared thermometers. A thermal imaging device would help identify areas of raised temperature (or ‘hotspots’) which others have reported to be indicative of pre-ulcerous inflammation [9, 10]. These could be missed during routine foot examination of the neuropathic diabetic foot, when signs and symptoms of inflammation are often lacking.

To address this need, a novel medical thermal imaging device was recently developed [15]. Laboratory testing showed that the overall temperature uncertainty of the thermal imaging device was ±0.2 °C (k = 2, 95% confidence limit) for the range 15 °C to 45 °C which is comparable to the uncertainty of the CE marked hand-held spot thermometers, (CE is abbreviated from Conformité Européenne, meaning European Conformity), [15]. The usefulness of this system in temperature assessment of the feet of healthy volunteers at 33 ROIs (12 plantar, 15 dorsal, 3 medial and 3 lateral) has been documented [16].

To assess the performance of the novel thermal imaging device in the assessment of foot temperatures in healthy volunteers in comparison with a hand-held infrared thermometer we selected five easily identifiable plantar foot landmarks (1st and 4th toes, 1st, 3rd and 5th metatarsal heads). The objectives of this study were twofold: firstly to measure the agreement in replication (three repeated measures) for the thermal imaging device and for the hand-held thermometer (inter-instrument agreement) and secondly, to measure the agreement between the thermal imaging device and the hand-held-thermometer (intra-instrument agreement) in the assessment of temperatures of the feet of healthy volunteers.

## Methods

### Participants

The study was carried out at three clinical centres as previously described [16]. Male and female volunteers were recruited if they had intact feet and no previous history of diabetes, foot ulcer or foot surgery either for correction of a foot deformity or following foot trauma. Subjects were excluded if they reported unsteadiness in gait, if they experienced burning pain, aching of the feet or legs, prickling sensation or numbness of the feet or legs or if they had any discomfort in the calf muscles when walking that was relieved with rest or any health problems affecting their feet and legs. The study was approved by London-City Road and Hampstead Research Ethics Committee (REC reference 15/LO/0070) and was carried out in accordance with the Declaration of Helsinki as revised in 2000. The study was registered on ClinicalTrials.gov website (Clinicaltrials.gov identifier NCT02317835). All subjects provided written informed consent and screening and assessment were performed at one study visit.

### Temperature measurement and data acquisition

Temperature measurements were carried with a novel thermal imaging device (Diabetic Foot Ulcer Preventions System, DFUPS), developed specifically for this investigation by Photometrix Imaging Ltd. in association with the University of South Wales [15, 16] and with a hand-held infrared thermometer (Thermofocus® 01500A3, Tecnimed, Italy). The thermal imaging device is a battery operated instrument with on-board software. The captured foot thermal image is downloaded on to a computer for further analysis [15]. Circles with an area equal to 1 cm^2^ are manually placed on ROIs of each foot. The Thermofocus is a non-contact spot thermometer, which measures the emitted thermal radiation of a selected ROI of the foot and converts that measurement into a temperature. The field of view of the scanned area is nominally 1 cm^2^.

Four thermal imaging systems (one for each clinical centre and one as a back-up) and four hand-held infrared thermometers (one for each clinical centre and one as a back-up) were used in the study. All devices were characterised at the National Physical Laboratory (NPL) before usage by the clinical centres, as described previously [15]. In brief, the thermal imaging systems and the hand-held infrared thermometers were evaluated to assess the temperature resolution, the spatial resolution and performance (repeatability, stability and accuracy). All devices were calibrated under laboratory conditions in terms of radiance temperature versus the NPL blackbody calibration sources [17] over the range of 15 °C to 45 °C, traceable to the international temperature scale of 1990 (ITS-90) with uncertainties of ±0.2 °C (*k* = 2, 95% confidence limit) quantified in accordance with the internationally agreed Guide to Uncertainty in Measurement (http://www.bipm.org/utils/common/documents/jcgm/JCGM_100_2008_E.pdf).

**Fig. 1:**
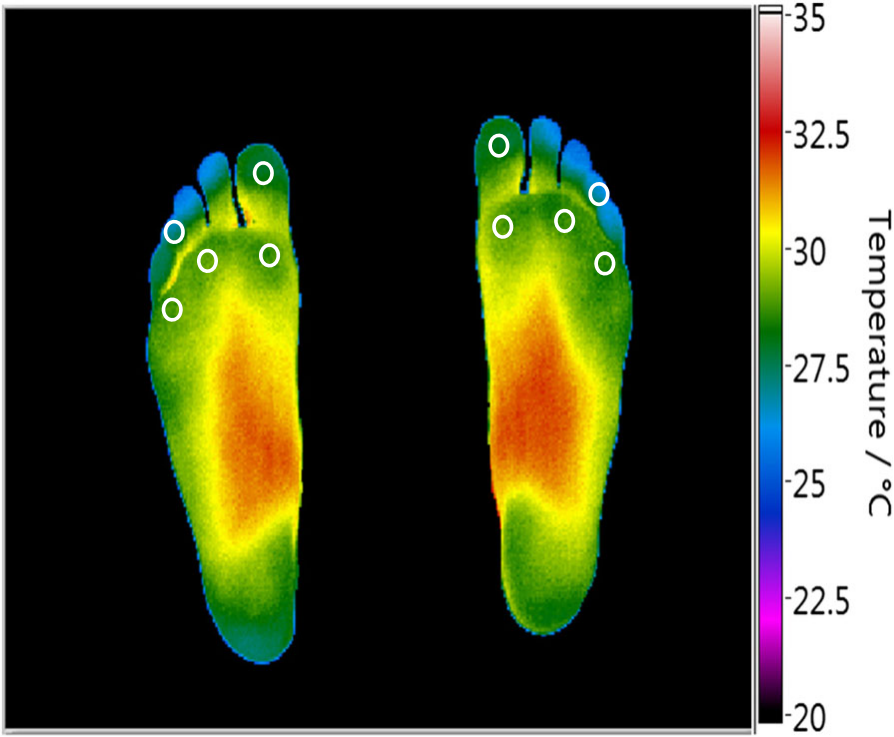
A typical example of a combined plantar thermal image of the right and left foot captured with the thermal imaging device in a healthy volunteer. The white circles show the manually selected ROIs

Participants were assessed after 20 min of barefoot rest on a podiatry chair with their legs extended and supported. Three consecutive measurement sequences were carried out. In each sequence, thermal imaging alternated with hand-held thermometry. Initially a combined plantar image of the right foot and left foot was captured with the thermal imaging device. This was followed by spot thermometry at five predefined ROIs (1st and 4th toes, and 1st, 3rd and 5th metatarsal heads). The temperatures of each ROI were measured with the hand-held thermometer initially on the right foot and then on the left foot. The same ROIs of the right foot and left foot were manually selected on each thermal image and the temperatures were recorded (Fig. 1).

### Statistical methods:

Temperature differences between feet (Right Foot-Left Foot) were calculated for each ROI for the thermal imaging device and for the hand-held thermometer, respectively. Each measure was replicated three times. The differences between repeated measurements as well as the differences between instruments (infrared thermal imaging device and hand-held thermometer) were modelled with multilevel linear regression and random effects analysis of variance. The agreement between the repeated measures at five ROIs for each instrument (intra-instrument agreement) and between the two instruments at the same ROI (inter-instrument agreement) was quantified using intra-class correlation coefficient (ICC) and the 95% confidence intervals (CI) following a multilevel modelling approach (random effects regressions). If a substantial agreement between replications was established, the replications within each ROI were averaged. Bland and Altman analysis and plots were used to complement the assessment of any bias between the two instruments [18]. The benchmark limits for agreement followed established classifications [18, 19]. In all cases, for more rigour, in addition to the point estimate, the lower limit of the 95% CI was taken into account.

## Results

A total of 105 subjects (52 males and 53 females; age range 18 to 69 years (mean age 44 ± 11 years (mean ± SD)), weight range 49 to 136 kg (mean weight 77.5 ± 16.2 kg), height range 1.50 to 1.98 m (mean height 1.70 ± 0.10 m), body mass index (BMI) range 18.2 to 51.8 kg/m^2^ (mean BMI 26.7 ± 5.4 kg/m2) were recruited in the study at the three clinical centres. Temperature measurements were carried out by trained operators (one operator per centre) and were taken in controlled room conditions. The mean study room temperature and humidity were 23 ± 0.5 °C (mean ± SD) and 50 ± 8%RH, (mean ± SD) respectively. In two subjects, the thermal imaging data was unavailable (the images were not saved after acquisition and could not be recovered). Repeated measurement data for both instruments were available for 103 subjects. The mean duration of the temperature assessment (three repeated sequences of alternating thermal imaging and hand-held thermometry) was 3 min ±40 s (mean ± SD). No correction was made for skin emissivity as only temperature differences were determined in this study and it was assumed that skin emissivity was the same at equivalent points on the foot.

### Intra-instrument agreement (agreement in replication)

The random effects linear regression analysis indicated that there were no significant differences in the temperature assessment between the three replications at all ROIs (1st toe *p* = 0.26; 4th toe *p* = 0.97; 1st metatarsal head *p* = 0.93; 3rd metatarsal head *p* = 0.69 and 5th metatarsal head *p* = 0.98). The intra-instrument agreement for the thermal imaging device and for the hand-held thermometer was similar at all five ROIs, as indicated by a non-significant replication-by-instrument interaction in any of the five measured ROIs: 1st toe *p* = 0.23; 4th toe *p* = 0.97, 1st metatarsal head p = 0.23, 3rd metatarsal head *p* = 0.84 and 5th metatarsal head *p* = 0.37.

The intra-instrument ICCs for the thermal imaging device ranged from 0.95 to 0.97 at the selected ROIs and the intra-instrument ICCs for the hand-held-thermometer ranged from 0.94 to 0.97, (Table 1).

**Table 1:** Intra-instrument agreement in repeated measures at five ROIs by instrument

ROIs	Hand-held thermometer	Thermal imaging device
1st toe	0.94 (0.92, 0.96)	0.95 (0.93, 0.96)
4th toe	0.95 (0.93, 0.96)	0.95 (0.94, 0.97)
1st metatarsal head	0.97 (0.96, 0.98)	0.97 (0.96, 0.98)
3rd metatarsal head	0.96 (0.94, 0.97)	0.96 (0.94, 0.97)
5th metatarsal head	0.97 (0.95, 0.98)	0.97 (0.96, 0.98)

Data are presented as ICC (95% C.I.) for each ROI by instrument

### Inter-instrument agreement between hand-held thermometer and thermal imaging device

Random effects linear regression, averaging the three replications at the selected ROIs indicated that the mean difference between instruments (hand-held thermometer minus thermal imaging device) ranged between − 0.01 °C and 0.21 °C and the inter-instrument ICCs ranged between 0.94 and 0.97, respectively (Table 2).

**Table 2:** Measure of agreement between hand-held thermometer and thermal imaging device at five ROIs

ROIs	Mean temperature difference (°C) between instruments^a^ (95% C.I.)	*P*-value	ICC (95% C.I.)
1st toe	0.04 (− 0.01, 0.10)	0.18	0.95 (0.93; 0.97)
4th toe	0.03 (− 0.05, 0.12)	0.42	0.94 (0.92; 0.96)
1st metatarsal head	−0.01 (− 0.05, 0.04)	0.81	0.97 (0.95; 0.98)
3rd metatarsal head	0.11 (0.05, 0.17)	< 0.001	0.96 (0.94; 0.97)
5th metatarsal head	0.21 (0.16, 0.27)	< 0.001	0.94 (0.91; 0.96)

^a^Hand-held thermometer minus thermal imaging device

**Fig. 2:**
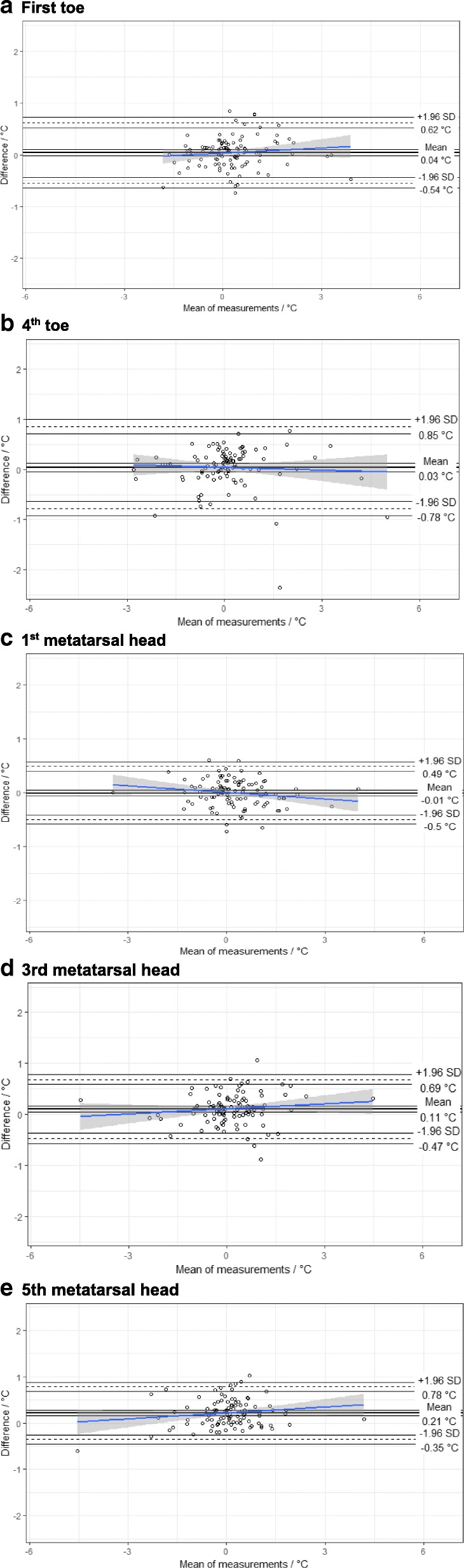
Bland and Altman plots of agreement between the thermal imaging device and the hand-held thermometer for the 1st toe (**a**), 4th toe (**b**), 1st metatarsal head (**c**), 3rd metatarsal head (**d**) and 5th metatarsal head

At all five ROIs, Bland and Altman analysis indicated that the mean differences between the two instruments were very close to zero (Table 3) and the Bland and Altman plots present the limits of agreement for all five ROIs (Fig. 2).

**Table 3:** Limits of agreement between hand-held thermometer and thermal imaging device at five ROIs

ROIs	Mean temperature difference (SD)^a^ °C	Lower Limit (95% C.I.) °C	Upper Limit (95% C.I.)°C
1st toe	0.04 (0.30)	− 0.54 (− 0.64 to − 0.44)	0.62 (0.52 to 0.72)
4th toe	0.03 (0.42)	−0.78 (− 0.93 to − 0.64)	0.85 (0.71 to 0.99)
1st metatarsal head	−0.01 (0.25)	− 0.50 (− 0.58 to − 0.41)	0.49 (0.40 to 0.57)
3rd metatarsal head	0.11 (0.29)	−0.47 (− 0.57 to − 0.37)	0.69 (0.59 to 0.79)
5th metatarsal head	0.21 (0.29)	− 0.35 (− 0.45 to − 0.26)	0.78 (0.69 to 0.88)

^a^Thermal imaging device - Hand-held thermometer

## Discussion

This study reports the performance of a novel thermal imaging device in the assessment of foot temperatures in healthy volunteers in comparison with a hand-held infrared spot thermometer.

Both instruments showed agreement in repeated temperature assessment and also agreement between instruments. Logistic regression analysis indicated that there were no differences in the repeated temperature assessment at five ROIs between the two instruments. The inter-instrument ICCs at all ROIs were equal to or above 0.95 for the novel thermal imaging device and equal to or above 0.94 for the hand-held infrared spot thermometer, indicating almost perfect agreement in replication by instrument. Moreover, there was substantial to perfect agreement in temperature assessment between the two instruments and the intra-instrument ICCs were equal to or above 0.94 at all five ROIs. Bland and Altman plots showed that only a few points were outside the limits of agreement. Based on the benchmark limits for agreement, these analyses demonstrated consistency of measure.

In addition to the reported non-inferior performance in temperature assessment at predefined ROIs, the novel thermal imaging device holds the potential to overcome the significant limitations of spot thermometry and provide an instantaneous thermal image of all sites of the feet (plantar, dorsal, lateral and medial views), [16]. Indeed, the advantages of a full imaging acquisition sequence including plantar, dorsal, medial and lateral views captured with DFUPS in the temperature assessment of the feet of healthy volunteers have been reported [16]. In addition, thermal imaging with DFUPS does not require any calibration for age, gender, weight, height or BMI and therefore it can be readily implemented in everyday clinical assessment. The importance of foot skin temperature monitoring in the identification of the early signs of inflammation has been emphasised in the 2015 guidelines of the International Working Group on the Diabetic Foot [20]. We have recently completed a multicentre clinical trial (NCT02579070) in high-risk diabetic foot patients to assess the usefulness of thermal imaging with DFUPS in addition to standard podiatric treatment to reduce diabetic foot ulcer recurrence. In addition to diabetic foot ulcer prevention, a further study is planned to investigate the usefulness of DFUPS in the assessment of the acute Charcot foot.

## Conclusions

The newly developed thermal imaging device showed very good agreement in repeated temperature assessments at defined ROIs as well as substantial to perfect agreement in temperature assessment with a hand-held infrared thermometer. This device fulfils the requirements of a reproducible and accurate thermal imaging device. It addresses the clinical need of a “portable, reliable and accurate” thermal imaging instrument [8, 13]. We believe that the developed thermal imaging device holds the potential of becoming a real asset in the diabetic foot clinic, to identify potential patients at risk of diabetic foot ulcer.

## Abbreviations

BMI: Body mass index
CE: is abbreviated from Conformité Européenne, meaning European Conformity
CI: Confidence intervals
DFUPS: Diabetic Foot Ulcer Preventions System
ICC: Intra-class correlation coefficient
NHS: National Health Service
NPL: National Physical Laboratory
REC: Research Ethics Committee
ROI: Region of interest
ROIs: Regions of interest

## References

[1] Boulton AJM, Vileikyte L, Ragnarson-Tennvall G, Apelqvist J (2005). The global burden of diabetic foot disease. Lancet.

[2] Edmonds ME (2004). The diabetic foot, 2003. Diabetes Metab Res Rev.

[3] Rice JB, Desai U, Cummings AK, Birnbaum HG, Skornicki M, Parsons NB (2014). Burden of diabetic foot ulcers for medicare and private insurers. Diabetes Care.

[4] Diabetes UK. Improving footcare for people with diabetes and saving money: an economic study in England. https://diabetes-resources-production.s3-eu-west-1.amazonaws.com/diabetes-storage/migration/pdf/Improving%2520footcare%2520economic%2520study%2520%28January%25202017%29.pdf Accessed 30 Apr 2018.

[5] Chammas NK, Hill RL, Edmonds ME. Increased mortality in diabetic foot ulcer patients: the significance of ulcer type. J Diabetes Res. 2016;2016:2879809. 10.1155/2016/2879809 PMC4860228 27213157

[6] Vamos EP, Bottle A, Edmonds ME, Valabhji J, Majeed A, Millett C. Changes in the incidence of lower extremity amputations in individuals with and without diabetes in England between 2004 and 2008. Diabetes Care. 2010;33(12):2592–7. 10.2337/dc10-0989 PMC2992196 20833865

[7] Ring F. Thermal imaging today and its relevance to diabetes. J Diabetes Sci Technol. 2010;4(4):857–62. 10.1177/193229681000400414 PMC2909517 20663449

[8] Pafili K, Papanas N. Thermography in the follow up of the diabetic foot: best to weigh the enemy more mighty than he seems. Expert Rev Med Devices. 2015;12(2):131–3. 10.1586/17434440.2015.990378 25483889

[9] Frykberg RG, Gordon IL, Reyzelman AM, Cazzell SM, Fitzgerald RH, Rothenberg GM, Bloom JD, Petersen BJ, Linders DR, Nouvong A, Feasibility NB. Efficacy of a smart mat Technology to predict development of diabetic plantar ulcers. Diabetes Care. 2017;40(7):973–80. 10.2337/dc16-2294 28465454

[10] Armstrong DG, Holtz-Neiderer K, Wendel C, Mohler MJ, Kimbriel HR, Lavery LA. Skin temperature monitoring reduces the risk for diabetic foot ulceration in high-risk patients. Am J Med. 2007;120(12):1042–6. 10.1016/j.amjmed.2007.06.028 18060924

[11] Houghton VJ, Bower VM, Chant DC. Is an increase in skin temperature predictive of neuropathic foot ulceration in people with diabetes? A systematic review and meta-analysis. J Foot Ankle Res. 2013;6:31. 10.1186/1757-1146-6-31 PMC3750703 23919736

[12] van Netten JJ, Prijs M, van Baal JG, Liu C, van der Heijden F, Bus SA. Diagnostic values for skin temperature assessment to detect diabetes-related foot complications. Diabetes Technol Ther. 2014;16(11):714–21. 10.1089/dia.2014.0052 25098361

[13] Bharara M, Cobb JE, Claremont DJ. Thermography and thermometry in the assessment of diabetic neuropathic foot: a case for furthering the role of thermal techniques. Int J Low Extrem Wounds. 2006;5:250–60. 10.1177/1534734606293481 17088601

[14] Prompers L, Huijberts M, Apelqvist J, Jude E, Piaggesi A, Bakker K, Edmonds M, et al. High prevalence of ischaemia, infection and serious comorbidity in patients with diabetic foot disease in Europe. Baseline results from the Eurodiale study. Diabetologia. 2007;50(1):18–25. 10.1007/s00125-006-0491-1 17093942

[15] Machin G, Whittam A, Ainarkar S, Allen J, Bevans J, Edmonds M, Kluwe B, Macdonald A, Petrova N, Plassmann P, Ring F, Rogers L, Simpson R. A medical thermal imaging device for the prevention of diabetic foot ulceration. Physiol Meas. 2017;38(3):420–30. https://doi.org/10.1088/1361-6579/aa56b1. 10.1088/1361-6579/aa56b1 28053300

[16] Macdonald A, Petrova N, Ainarkar S, Allen J, Plassmann P, Whittam A, Bevans J, Ring F, Kluwe B, Simpson R, Rogers L, Machin G, Edmonds M. Thermal symmetry of healthy feet: a precursor to a thermal study of diabetic feet prior to skin breakdown. Physiol Meas. 2017;38(1):33–44. https://doi.org/10.1088/1361-6579/38/1/33. 10.1088/1361-6579/38/1/33 27941234

[17] Machin G, Chu B. High-quality blackbody sources for infrared thermometry and thermography between-40 and 1000 degrees C. Imaging Sci J. 2000;48(1):15–22.

[18] Bland JM, Altman DG. Statistical methods for assessing agreement between two methods of clinical measurement. Lancet. 1986;1:307–10. 2868172

[19] Landis JR, Koch GC. The measurement of observer agreement for categorical data. Biometrics. 1977;33:159–74. 843571

[20] IWGDF Guidance on the prevention of foot ulcers in at-risk patients with diabetes. http://www.iwgdf.org/files/2015/website_prevention.pdf. 10.1002/dmrr.2696 26334001

